# Pulsed field ablation in medicine: irreversible electroporation and electropermeabilization theory and applications

**DOI:** 10.2478/raon-2025-0011

**Published:** 2025-02-27

**Authors:** Edward J Jacobs, Boris Rubinsky, Rafael V Davalos

**Affiliations:** 1 Wallace H Coulter School of Biomedical Engineering, Georgia Institute of Technology & Emory Medical School, Atlanta, Georgia, USA; 2 Department of Bioengineering and Department of Mechanical Engineering, University of California, Berkeley, Berkeley, California, USA

**Keywords:** puled-field ablation, irreversible electroporation, pulsed electric fields, margin accentuation, oncology, atrial fibrillation

## Abstract

**Background:**

Focal ablation techniques are integral in the surgical intervention of diseased tissue, where it is necessary to minimize damage to the surrounding parenchyma and critical structures. Irreversible electroporation (IRE) and high-frequency IRE (H-FIRE), colloquially called pulsed-field ablation (PFA), utilize high-amplitude, low-energy pulsed electric fields (PEFs) to nonthermally ablate soft tissue. PEFs induce cell death through permeabilization of the cellular membrane, leading to loss of homeostasis. The unique nonthermal nature of PFA allows for selective cell death while minimally affecting surrounding proteinaceous structures, permitting treatment near sensitive anatomy where thermal ablation or surgical resection is contraindicated. Further, PFA is being used to treat tissue when tumor margins are not expected after surgical resection, termed margin accentuation. This review explores both the theoretical foundations of PFA, detailing how PEFs induce cell membrane destabilization and selective tissue ablation, the outcomes following treatment, and its clinical implications across oncology and cardiology.

**Conclusions:**

Clinical experience is still progressing, but reports have demonstrated that PFA reduces complications often seen with thermal ablation techniques. Mounting oncology data also support that PFA produces a robust immune response that may prevent local recurrences and attenuate metastatic disease. Despite promising outcomes, challenges such as optimizing field delivery and addressing variations in tissue response require further investigation. Future directions include refining PFA protocols and expanding its application to other therapeutic areas like benign tissue hyperplasia and chronic bronchitis.

## Electropermeabilization theory

**FIGURE 1.**
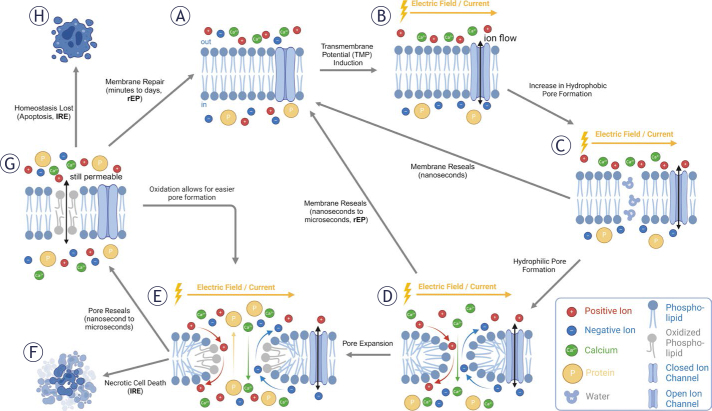
Conceptual schematic of the molecular mechanisms of electropermeabilization. **(A)** An intact cell membrane **(B)** in an exogenous electric field experiences an induced transmembrane potential. **(C)** Hydrophobic pores become energetically favorable as water infiltrates the bilayer. With the removal of the applied electric field, the hydrophobic pore reseals within nanoseconds. **(D)** If higher and longer external electric fields are applied, phospholipids invert to form small hydrophilic pores that allow the passage of ions and small molecules. Elastic forces within the membrane allow for these pores to reseal within nanoseconds to microseconds after the removal of the electric field. **(E)** With higher magnitude and longer duration electric fields, pores number may increase, and nucleated pores may expand or combine, allowing the transport of larger molecules and higher quantities across the membrane. Significant lipid oxidation is indicated to occur at high electric fields. **(F)** If excessive, the lipid bilayer may hemorrhage leading to lytic (necrotic) cell death. **(G)** After cessation of the applied electric field, the cell membrane may remain permeable due to the presence of lipid oxidation, which, in return, also allows for easier pore formation upon the introduction of another electric field. **(H)** As significant mass transport occurs over the cell membrane, the cell may lose homeostasis and die through regulated cell death, or **(G)** the cell may repair the permeable and damaged cell membrane to regain homeostasis.

**Figure 2.**
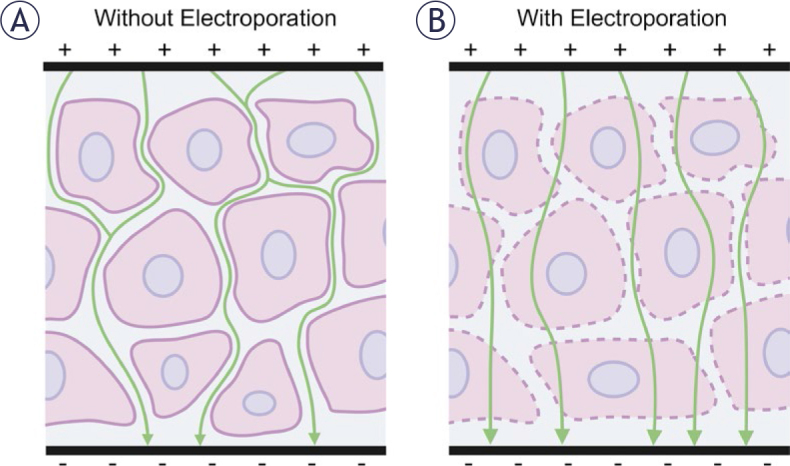
Electric field and current through heterogeneous tissue. **(A)** Without electroporation, current (green arrows) passes around the cells (pink) through the extracellular space (blue). **(B)** Electroporation allows for current to pass through the cells, but it is still influenced by tissue heterogeneity.

Electropermeabilization is a biophysical phenomenon in which exogenous electric fields (EFs) increase the permeability of the cellular membrane (Figure 1). The application of an electric potential across tissue generates an EF whose shape and magnitude depend on the local electrical tissue properties. The EF induces ion movement (i.e., current) within the tissue (Figure 2), and the subsequent charge concentration around cells generates an electric potential across the cellular membrane. This transmembrane potential (TMP) permeabilizes the cellular membrane through phospholipid oxidation^1–6^, modulation of electrically-induced proteins^7^, and the generation of nano-scale pores (electroporation).^8^ Standard electroporation theory and experiments suggest that pores are the dominant factor in mass transport across the membrane following electropermeabilization^9^ and that pore formation occurs when the induced TMP exceeds a critical threshold (~0.258 V).^10^ The magnitude of the induced TMP is dependent on the local geometry of the membrane and directly related to cell size and shape.^11,12^ Once the exogenous EF is removed, the hydrophobic interactions, Van der Waals forces, and electrostatic interactions within the phospholipid bilayer may cause the pores to reseal within seconds to hours.^13–15^ The transitory formation of pores is called reversible electroporation (rEP) and has been used for decades to deliver chemotherapeutics (electrochemotherapy; ECT)^16–18^, calcium (calcium electroporation; CaEP)^19–25^, genetic material (gene electrotherapy, GET)^26,27^, and otherwise impermeable substances^28^ into cells. With the application of higher magnitude and protracted pulses, pore nucleation increases within the cellular membrane, and existing pores expand, allowing for increased mass transport, consequently with the increased likelihood of losing homeostasis or causing cellular membrane hemorrhage.^7,29,30^

Concomitant to pore formation, the applied EF generates reactive oxygen species (ROS) that can induce lipid oxidation within the membrane.^1–6^ Lipid oxidation increases the spacing between lipids and decreases membrane thickness, leading to increases in membrane permeability and electrical conductivity.^5,6,31^ Since oxidative agents are slowly removed from the membrane^32^, these effects also persist after pores reseal.^4,5^ Further, subsequent pore formation and increased oxidation may occur more easily at locations of previous oxidation^33^, and oxidative lipids may diffuse throughout the membrane between applied pulses.^34^ Excessive oxidation can occur using higher magnitude EFs, longer pulses, and more pulses^2–4^, leading to complete bilayer disruption and cell death.^31^

Further, PEFs can destabilize and fragment cytoskeletal elements^35^, including actin filaments^36–39^, microtubules^40,41^, and intermediate filaments^41–43^, which collectively maintain cell shape, enable intracellular transport, and support membrane stability.^44^ The membrane and cytoskeleton are functionally and structurally linked, so disruption can exacerbate membrane deformation and impair cellular mechanical properties, increasing the susceptibility of the membrane to subsequent pore formation and enhancing ion and molecule transport.^39,45^ Cytoskeletal disruption may also interfere with cellular signaling pathways reliant on cytoskeletal integrity, affecting processes such as cell adhesion, motility, and division^36,42,45^, with implications in blood vessel permeabilization.^46–48^ As with membrane oxidation, cytoskeletal damage can persist even after the EF is removed, leading to prolonged changes in cell structure and negatively impacting cell viability and function.^49,50^

## Pulsed field ablation techniques in medicine

Irreversible electroporation (IRE) was initially considered the upper limit of rEP and, as such, something to be avoided when post-treatment viability is desired.^11^ With their seminal paper, R. Davalos, L. Mir, and B. Rubinsky mathematically described that EFs necessary to induce clinically relevant volumes of IRE did not simultaneously generate significant Joule heating and subsequent thermal damage.^51^ Edd *et al*. supported this hypothesis by generating contiguous ablations in rat livers at EFs indicated to not cause thermal damage.^52^ Following, Al-Sakere *et al*. reported the first successful use of IRE in oncology, achieving complete regression in 92% (12/13) of treated cutaneous mouse tumors using an optimized waveform (80 monophasic pulses of 100 μs at 0.3 Hz and 2500 V/cm), with a maximum measured temperature of 37.5°C.^53^ The results from these studies demonstrated the feasibility of increasing the number of pulses from conventional ECT (8 pulses) without inducing thermal damage and provided the foundation for parameters used in current IRE protocols.

**FIGURE 3.**
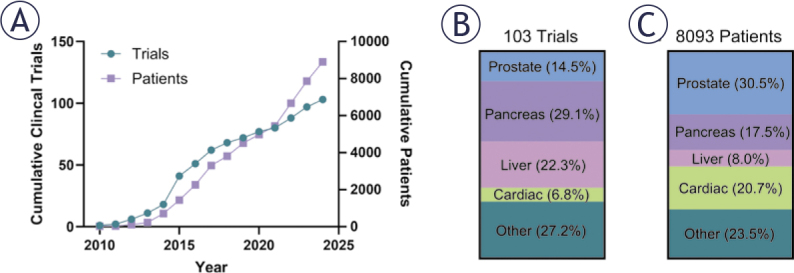
**(A)** cumulative registered patient and trial numbers for IRE and PFA on ClinicalTrials.gov. **(B)** Breakdown of trials and **(C)** patient populations by tissue type. Other contains renal, lung, stomach, esophageal, gallbladder, hilus pulmonis, extremity, lymph node, intestinal, rectal, laryngeal, head and neck, and breast cancers; benign prostate hyperplasia; chronic bronchitis; tonsillar hypertrophy.

Shortly after, Bertacchini *et al*. developed the first IRE generator approved for clinical use.^54^ Since the introduction of IRE in the clinic in 2010, over 100 clinical trials have been registered worldwide (Figure 3), with hundreds of research articles published demonstrating safe and effective treatment of prostate^55–64^, pancreas^65–74^, liver^75–84^, and kidney^85–97^ tumors, but feasibility in many other solid tumors like lung^98–99^ and brain^100^ has been demonstrated.

**FIGURE 4.**
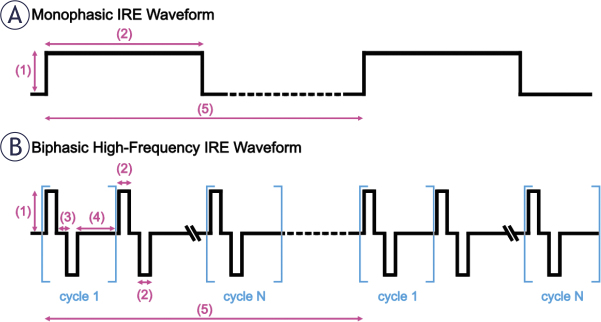
**(A)** Monophasic IRE waveform and **(B)** Biphasic H-FIRE waveform. (1) Magnitude (voltage or current), (2) pulse width, (3) interphase delay, (4) interpulse / intercycle delay, (5) burst repitition interval.

IRE as a clinical technique is described as a non-thermal focal ablation modality that employs high-magnitude (1–3 kV) and short (70–100 μs) monophasic pulses (Figure 4A) generated between conductive electrodes placed into or around the targeted tissue. In clinical practice, conventional monophasic IRE pulses must be delivered using general anesthesia and prophylactic neuromuscular blockers to reduce muscle contractions.^53,101–103^ Induced muscle contractions are undesirable in debilitated patients and can cause an involuntary shift in the electrode locations, leading to incomplete ablation of the target region or puncture of neighboring critical structures (e.g., blood vessels, nerves). Early experience with IRE was also associated with incidence of cardiac dysrhythmia, so pulse delivery is now synchronized to the R-wave on electrocardiogram (ECG) recording with a 0.05 s delay to avoid interference with normal cardiac rhythm.^104^ IRE is still contraindicated in patients with cardiac arrhythmia, as pulses cannot be consistently synchronized with the cardiac refractory period.

To overcome these limitations, Arena *et al*. developed High-Frequency IRE (H-FIRE)^105^, which utilizes a series of short (0.5–10 μs) biphasic pulses. The H-FIRE waveform is constructed of a positive pulse, interphase delay (d1), negative pulse, and interpulse delay (d2), repeated for several cycles to achieve an on-time comparable to IRE (Figure 4B). H-FIRE significantly reduces muscle contraction during treatment and obviates the use of neuromuscular blockers or cardiac synchronization.^106^ Further, the shorter pulses are suggested to provide more predictable ablations when the pulse width is below the cell membrane charging time of 1–2 μs.^107–108^ However, as a consequence of the reduced membrane charging, the EF threshold (EFT) required to induce electroporation increases as pulse width decreases, but thermal heating remains relatively the same.^109–110^ H-FIRE has been used pre-clinically to treat breast^111^, liver^106^, brain^112^, lung^113^, and prostate^114^ cancer with mixed results. To date, H-FIRE has not demonstrated the same tumor ablation capability as IRE, but H-FIRE has been evaluated clinically in prostate cancer, offering a potential reduction in experienced complications.^115,116^ Notwithstanding, H-FIRE has gained prodigious attention for the treatment of cardiac arrhythmias under the name PFA.^117–129^ Between the different groups, H-FIRE (i.e., PFA) is indicated to have been performed in over 100,000 patients as of September 2024, not without appropriate criticism of the lack of transparency for treatment details.

Since PFA primarily induces cell death through permeabilization of the cell membranes, the PEFs minimally affect proteinaceous structures. The nonthermal mechanism is paramount for the control of diseased tissue near critical structures, such as bowels^97^, ducts^130^, mature blood vessels^131,132^, esophagus^133^, and nerves^56,134,135^, where surgical resection and thermal ablation methods are contraindicated. Further, PFA is not influenced by the “heat sink” effect, where blood flow in adjacent vessels dissipates heat, reducing ablation effectiveness and potentially sparing targeted tissue. This allows PFA to completely treat tissue abutting blood vessels. Narayan *et al*. examined the patency of 158 vessels with a mean distance from the treatment lesions of 2.3 mm and noted abnormal changes in 4.4% (7/158) of vessels.^132^ Only 1.4% (2/158) were hemodynamically significant, with many vessels that experienced thrombosis post-treatment already heavily involved before treatment. Tumors abutted 40 vessels and encased 10 vessels, but 96% (48/50) maintained patency despite being directly within the ablation. Further, Li *et al*. found that neurovascular bundles are not destroyed even when directly treated with ablative PEFs.^134^ Subsequent studies have observed that there may be some degree of thermal damage to the tissue immediately near the treatment electrodes^136–137^, so careful planning and probe placement are still needed.

## Pretreatment planning

**FIGURE 5.**
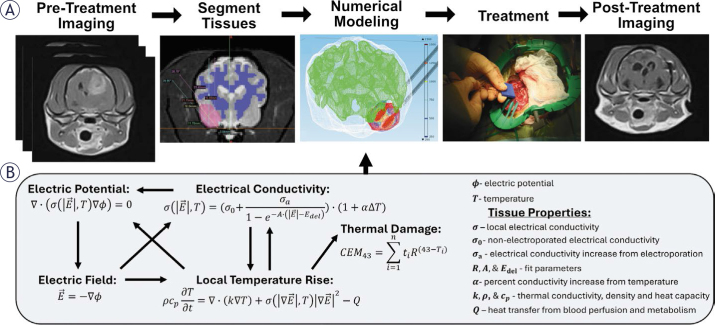
Pulsed field ablation treatment planning pipeline. **(A)** Images of the region of interest are taken through CT, MRI, US, or other modalities. Typically, segmentation is performed to define individual tissue regions before computational modeling, as the dynamic electric field, temperature, and conductivity distributions are tissue dependent. Numerical modeling is performed with the intent to maximize targeted tissue coverage with a critical electric field while minimizing deleterious effects on nearby structures. Following, the protocol is implemented for the treatment of the target tissue. While computational modeling can inform treatments, the exact application of PFA can often differ from *a priori* computational modeling. Post-treatment imaging is frequently used to assess acute and long-term ablation success. **(B)** Tissue electroporation modeling is multifaceted and requires knowledge about multiple electroporation-dependent and electroporation-independent tissue properties. The local electric potential depends on the local electrical conductivity and temperature. The electrical conductivity also depends on the local temperature and the electric field, as pore formation due to the local electric field allows for current to flow through cells. Subsequently, temperature generation depends on the electric field magnitude and the local conductivity. Images in panel A were adapted from various sources for instructional purposes.^100,235^

Computational modeling is necessary for the successful delivery of PFA^138^, as the entire target tissue must be covered by a critical EF while minimizing collateral damage to nearby critical structures.^139^ Treatment planning includes (Figure 5):

### 1. Imaging of the treatment area and surrounding structures

Before surgery, the location, size, and geometry of the tissue to be treated are determined with one or more imaging modalities, including contrastenhanced computed tomography (Ce-CT), positron emission tomography (PET), magnetic resonance imaging (MRI), and 3D-mapping biopsy for prostate cancer (PCa). Except for PCa, Ce-CT is the most used modality due to its availability, high resolution, and ability to rapidly create multi-planar reconstructions of the tumor and surrounding structures.^140^ For cancer patients, tumor growth or shifting may cause differences between prior- and intra-procedural images, so Ce-CT also allows for rapid adjustments in the treatment planning and probe position.^140–142^

### 2. 3D reconstruction of the anatomy of the treatment area

Multi-planar images are imported into a segmentation software (e.g., 3DSlicer) to separate the tumor, parenchyma, and nearby structures. The geometries are then meshed for importing into finite element analysis software (e.g., COMSOL™).

### 3. Define the electroporation-dependent material properties for the different tissues

*A priori* information about the target tissue is needed for accurate treatment modeling. Both the EF and temperature distributions strongly depend on the tissue-specific electrical properties^143,144^, which both differ between patients in healthy and malignant tissues and change non-linearly from the electroporation process itself.^145^ Results in computational modeling significantly differ when considering electroporation effects^146,147^, but validated tissue properties are sparse within the literature.

Conventional methods for tissue characterization use *ex vivo* tissue slices with fixed geometries to translate impedance at different applied EF magnitudes to conductivity.^146,148^ Quantifications are often limited to healthy animal tissues due to their availability and can misrepresent the targeted tissue, especially when translating results to tumors. Tissue characterization using patient-derived xenografts is more representative^149^, but they can take weeks to grow, are not widely available during treatment planning, and do not replicate *in situ* conditions. Further, even within a specific tumor type, there can be a high degree of tumor tissue heterogeneity between patients and even between tumors at different locations in the body. Translating experimentally found properties to an individual can be unreliable, so improved methods for patient-specific tissue characterizations are greatly needed.^147^

In addition to simulating the EF and thermal distributions, it is necessary to know the EFT of the tissues being treated to quantify the lesion coverage. Values for the lethal EFT are variable within the literature due to the lack of validated and standardized protocols. Thresholds gathered *in vitro* using cuvette systems are typically higher than those gathered using 2D or 3D platforms, but *in-situ* data is the most translatable.^150^ Pulse widths from nanoseconds to milliseconds will generate ablations, but pulse width negatively correlates with EFTs.^109,110,151,152^

### 4. Incorporation of treatment probes within the model and numerical optimization

Intrinsic tissue properties cannot often be changed; thus, treatment parameters (i.e., voltage, probe geometry, and PFA waveform) must be adjusted to find solutions that solve the desired objective. The two main objectives that are usually investigated for PFA are (1) encompassing the target tissue with a lethal EF while (2) minimizing Joule heating and subsequent thermal damage to nearby critical structures.

The number of probes depends on the ability to cover the tumor and margin with a lethal EF. For deep soft tissue neoplasms, typically 2 to 6 monopolar probes are inserted into or around the neoplasm. For lesions smaller than 2 cm, 3 probes are placed at the periphery of the tumor in a triangle; for lesions between 2–3 cm, 4 probes are placed at the periphery in a square; for lesions larger than 3 cm, 4–6 probes are used, with 1–2 of the probes placed within the lesion and the rest at the periphery.^153^ The distance between electrode pairs should not exceed 2.2 cm, but values have ranged from 0.7 to 2.9 cm in literature. The electrode exposure can vary from 0.5 to 3 cm, but 1.5 cm is the most common. The applied current scales linearly with electrode exposure, and too large of an exposure can trigger the overcurrent on electroporation generators at 50 A. Therefore, if the target is larger than the possible electrode exposure, the deepest portion of the target should be treated first; then, the electrodes can be “pulled back” for subsequent treatments to ensure overlapping and cohesive ablations.

Applied EFs or “voltage-to-distance ratios” (VDRs) typically range from 1200 V/cm to 2000 V/cm for IRE and 2000 V/cm to 3000 V/cm for H-FIRE. Higher VDRs will generate larger ablations at the consequence of increased Joule heating, neuromuscular excitation, and electrochemical effects.

## Probe positioning and treatment

IRE has been successfully performed through intraoperatively^154^, laparoscopy^155^, and percutaneous^156^ insertion of treatment probes. For percutaneous insertion, the probes must be carefully inserted under contrast-enhanced ultrasound (ce-US) or ce-CT guidance to prevent puncturing sensitive structures and maintain parallel insertion of the electrodes. Imaging is used to verify correct probe placement and measure the center-to-center probe separation to calculate the VDR. Probes should be placed parallel to each other with no more than 10-degree deviations to prevent irregular ablations and possible incomplete treatment.

Despite the EF coverage ultimately dictating ablation size, clinicians have found that electrical currents between 20 and 40 A during IRE provide sufficient ablations. With the NanoKnife system, 10 pulses are initially delivered to assess the applied current between each electrode pair. Following, if the current is adequate, the rest of the treatment will be delivered. Otherwise, the clinicians will increase or decrease the VDR to achieve the desired current and then deliver the appropriate number of pulses. An applied potential is only generated between one electrode pair at a time, and the final train of pulses is typically either 70 or 90 pulses between each probe pair.

In addition to cardiac arrhythmia, other absolute contraindications for PFA include the presence of non-removable pacemakers or implantable cardioverter defibrillators, a history of epilepsy or seizures, a history of bleeding disorders, and the presence of anatomical obstacles blocking safe probe insertion.

## Post treatment imaging

CT imaging is predominantly used after the procedure to determine treatment success and to evaluate disease recurrence or remission during follow-ups^157^, but ablations are also regularly visualized using PET^140^, MRI^157^, and US.^137^ Further, both IRE and H-FIRE produce ablations with sharper delineation than other ablation modalities.^158^ Histology of ablations demonstrates demarcation between the ablated and live tissue on the order of 1–2 cells.

## Cell death and immune activation

Given the complex and nuanced processes involved, the cell death mechanisms following IRE and H-FIRE are still under investigation. Researchers originally attributed necrosis due to disruption of the osmotic balance as the killing mechanism of electroporation. However, in the late 1990s, it was demonstrated that electroporation not only caused necrosis but also induced delayed cell death following chromosomal DNA fragmentation, which is an explicit indication of late apoptosis.^159,160^

There is a plethora of competing findings for cell death pathways and mechanisms following PFA, including immunogenic (e.g., necrosis, necroptosis, and pyroptosis) and non-immunogenic (e.g., apoptosis) cell death.^136,159–163^ Each pathway has unique implications for treatment side effects, immune activation, and efficacy.^164^ Increasing evidence suggests that H-FIRE induces delayed, regulated cell death while IRE induces immediate, lytic cell death.^163,165,166^ Further, it is suggested that higher EFs are more likely to induce necrosis through membrane hemorrhaging and thermal damage, while lower EFs may permit membrane recovery but induce regulated cell death following ROS generation, DNA damage, mitochondrial damage, ATP loss, osmotic imbalance, or calcium influx.^29,136,165,166^ While apoptosis is frequently highlighted as a key form of cell death in PFA, immediate cell death observed following IRE and H-FIRE often shows characteristics of necrosis. Thus, rather than a single pathway, it is likely a combination of overlapping death mechanisms that lead to the loss of cellular homeostasis.

### I. PFA reduces the anti-inflammatory cell populations within the tumor microenvironment

**FIGURE 6.**
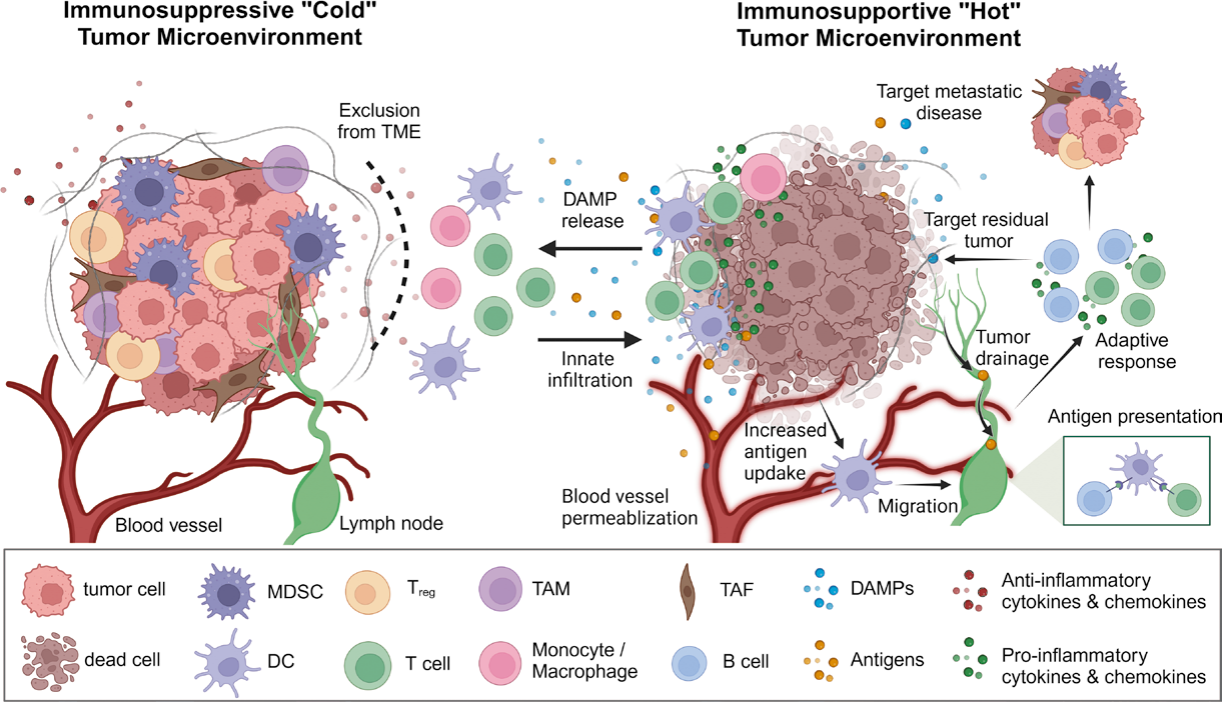
Immune response following pulsed field ablation. The tumor microenvironment (TME) evolved through all stages of cancer progression and protects itself through reprogramming immune cells (T regulatory cells [T-reg], myeloid-derived suppressor cells [MDSCs], and tumor-associated macrophages [TAMs]), attracting stromal cells (endothelial cells and fibroblasts) that help deposit a dense extracellular matrix (ECM). This produces an immunosuppressive “cold” tumor that excludes normal immune cells from infiltrating. Pulsed-field ablation indiscriminately kills tumor cells, stromal cells, and immunosuppressive immune cells within the ablation and restructures the ECM. The removal of active immunosuppression, permeabilization of mature blood vessels, and release of Damage Associated Molecular patterns (DAMPs) by IRE entices innate immune cell infiltration. Tumor antigens are released by treated cells, which are either taken up by dendritic cells or drained directly into lymph nodes for antigen presentation. Tumor-specific T- and B-cells mature within the lymph nodes, then antigen-specific T- and B-cells leave the lymph node to potentially remove residual cancer or target distant metastatic disease.

In many solid tumors, multiple cell populations contribute to the immunosuppressive “cold” TME (Figure 6), including differentiated cancer cells, cancer stem cells, tumor-associated fibroblasts (TAFs), and immunosuppressive immune cells (ISICs) (e.g., tumor-associated macrophages [TAMs], myeloid-derived suppressor cells [MDSCs], and regulatory T-cells [T_reg_]).^167^ Further, the epigenetic and cellular composition of tumors can vary between patients, between different tumors within a patient, and even at different locations within the same tumors^168^, making it challenging to provide single-target therapeutics. PFA acts indiscriminately on proliferating and non-proliferating cells^169^ within the critical EFT. Therefore, recalcitrant (e.g., cancer stem cells^170,171^) and immunosuppressive cells (TAMs, MDSCs, TAFs, and T_reg_^111,172,173^) are removed in addition to bulk tumor cytoreduction.

### II. PFA effectively reverses the stromainduced immunosuppression

PFA ablation alters the physical properties of the TME through reduction of the extracellular matrix density and rigidity^174,175^ and increases tumorassociated blood vessel permeability.^47,48,137,175,176^ These both reduce tumor-associated hypoxia that impedes leukocyte function.^175^ Increases in microvascular density are indicated after treatment^174,175^, but this may be attributed to transient decreases in vascular junction integrity and subsequent increases in the expression of junction proteins to regain microvasculature function. The preservation of mature vasculature patency while increasing permeability allows for infiltration of leukocytes and transport of TAAs to tumor-draining lymph nodes^238^. These results are not replicated in other focal ablation therapies, indicating that IRE may uniquely modulate the TME. Regeneration of the ablation site by parenchymal cells is also indicated at 1–2 weeks post ablations^177^, but underlying tissue disease or chemoembolization may prevent the healing process.^95^

### III. PFA induces a pro-inflammatory TME and activates the adaptive immune system

In addition to reducing anti-inflammatory cell populations, PFA actively promotes an immunesupportive TME. Damage associated molecular patterns (DAMPs) are released by electroporated cells and recognized by the innate immune system for generating early inflammation.^111,175^ Tumorassociated antigens (TAAs) are also released and evaginated by dendritic cells and macrophages for antigen presentation.^178^ Unlike with thermal ablation modalities, DAMPs and TAAs released by electroporated cells are presumably not destroyed due to the lack of sufficient thermal heat to denature proteins, potentially allowing for the priming of mature T-cells with receptors directed at the *insitu* protein motif.^179^

Although PFA treatment success is not predicated by the induction of an anti-tumor immune response, both *in vivo* and clinical data suggest a correlation between immune activation and progression-free survival (PFS) and overall survival (OS). He *et al*. demonstrated the disparity in patient OS when gating by immune activation; when separating patients into high and low T-lymphocyte responses, there was 70–80% and 0% survival at 30 months post-IRE, respectively.^74^ Goboers *et al*. found that T-cell activation correlated with pretreatment tumor sizes and suggested that antigen release may correlate with the extent of ablation.^173^ Larger ablation volumes would presumably induce more TAA and DAMP release while generating a larger variety of cell death mechanisms to create a robust immune response. They also found a decrease in circulating dendritic cell populations indicative of activation-induced migration to lymph nodes and treated tissue, which was supported by the activated T-cells expressing specific receptors against prostate cancer-associated antigens.

### IV. PFA can be combined with immunotherapies

To consistently generate persistent peripheral antitumor immune activation, current research aims to adjust pulsing waveforms to generate more inflammatory cell death modalities or combine treatment with adjuvant immunotherapies. The combination of IRE and immune checkpoint inhibitors (ICIs), such as anti-CTLA4, anti-PDL1, and anti-PD1, have positive results in both mice and humans.^172,175,178,180^ He *et al*. presented promising results when combining IRE with anti-PD1 in Stage III locally advanced pancreatic cancer, achieving an overall survival of 44.3 months versus 23.4 months for IRE alone.^180^ Further, they did not observe differences in adverse side effects between the two treatment groups, demonstrating that ICIs may offer a significant increase in IRE efficacy without additional side effects. Primary tumor ablation with IRE in a PCa mouse model, followed by anti-CTLA4 and anti-PD1 immune checkpoint inhibitors, induced a significant increase in both tissue-resident and circulating memory cytotoxic T-cells with T-cell receptors targeting PCa-specific antigen, SPAS-1.^178^ Subsequently, this work indicated that a tumor vaccine effect was achieved by the tissue-resident and circulating memory cytotoxic CD8+ T-cells, limiting the reintroduction of new PCa. A recent direct comparison of IRE with cryoablation (CA) and thermal ablation further demonstrated that anti-PD1 synergizes best with IRE, leading to longer tumor-free survival, increased infiltration of CD8+ T-cells, and protection against tumor reintroduction.^181^ Due to the modulation of the immunosuppressive TME, the efficacy of dendritic cell vaccination is improved after IRE.^182^

Despite promising results, local and distant tumor recurrence still occurs. A potential reason for the eventual tumor recurrence is that major histocompatibility complex I (MHC I) downregulation occurs 30–100% in many cancer types, with pancreatic cancer having a suppression rate of 40–100%.^183,184^ IRE clearly benefits from an induced immune response, but without antigen presentation for T-cell recognition, the local and metastatic micro-tumors are hidden from the heightened immune response and eventually repopulate local and distant sites. Lin *et al*. demonstrated the potential for combining IRE with autologous γδ T-cells, which can recognize and lyse cancers in an MHC-unrestricted manner. Patient γδ T-cells were isolated from the blood, expanded, and then reintroduced after IRE through at least 2 cycles.^185^ Patients with multiple infusions survived longer after treatment (17 months) than patients with a single infusion (13.5 months) or IRE alone (11 months). Further, IRE has been combined with natural killer (NK) cells^186–189^, which recognize cells that have downregulated MHC I receptors.^190^ Despite only evaluating the efficacy at 1-mo post-treatment, a randomized study of 92 LAPC patients found that the IRE-NK group achieved an overall response of 71.7% compared to IRE alone with 56.5%.^188^

## Prostate (PCa)

PCa is a leading cause of cancer-related deaths among men^191^, and the contemporary treatment for localized PCa is active surveillance, radical prostatectomy, and radiation therapy. Routine prostate examinations are becoming increasingly popular, resulting in earlier detection of manageable small-volume neoplasms. While whole-gland approaches have historically offered the best possible oncological outcome for local disease, low- to intermediate-risk patients may not benefit from radical treatments, as damage to the neurovascular bundle, external sphincter, bladder neck, urethra, and rectum are often associated with gastrourinary dysfunction which could include impotence, incontinence, pain, loss of rectal control, and loss of sensation. IRE offers a valuable treatment option for these patients, as the negative side effects can be circumvented while still achieving sufficient oncological outcomes. Further, IRE can be successfully delivered to any region of the gland (apex, middle, or base) with similar disease control^192^, while other focal ablation therapies are known to be preferential for certain areas.^193,194^

The first evaluation of IRE in the prostate was performed by Onik *et al*. in 2007 in six healthy canine prostates.^61^ Histology revealed a fine demarcation between the unaffected and necrotic prostate tissue, spanning only a few cells. When directly including the urethra within the ablation, necrotic glandular tissue abutted urethral structures without necrosis within the sub-mucosa. Vessel patency was also preserved when deliberately treating the neurovascular bundle, though variable endothelial and fibrinoid necrosis was observed. The authors expressed that nerves within the neurovascular bundles did not appear to be affected, with no evidence of ganglion cell death. Following, Onik *et al*. performed the first human clinical trial for IRE, involving 16 patients with low- to moderate-risk prostate cancer in a series of outpatient procedures.^62^ All patients were continent immediately after IRE, and all patients who were potent before the procedure were still potent after the procedure. Two patients who had bilateral areas treated required 6 months for a full return of potency. Color Doppler US showed intact flow within the neurovascular bundle immediately after the procedure, and postoperative biopsies taken from the area of previously known cancer in 15 patients showed no evidence of cancer.

A disadvantage of focal ablation therapies is the possible presence of multi-focal disease that is not initially diagnosed through imaging or biopsy. As PCa is frequently multi-focal, IRE application to multiple segments or the entire prostate gland can extend its coverage. A multi-center randomized clinical trial evaluated the control of focal and extended IRE in 106 low- to intermediate-risk patients.^56^ A similar total rate of recurrence was observed, but the extended ablation cohort experienced lower recurrence away from the lesion site. Guenter *et al*. also presented encouraging results from a large retrospective assessment of 429 patients with low (n = 25), intermediate (n = 88), and high-risk (n = 312) prostate cancer.^195^ Patients were treated focally (n = 123), sub-whole-gland (n = 154), whole gland (n = 134), or for recurrent disease after previous treatment with other modalities (n = 63). During a maximum follow-up time of 72 months, 3 (12%), 18 (20.4%), and 26 (8.3%) recurrent cancers were observed in the low-, intermediate-, and high-risk groups, respectively. Urinary continence was preserved in all patients. Ten patients developed a temporary decrease in erectile function, with 4 patients experiencing a decrease longer than a year. Scheltema *et al*. recently released their longer-term (60 months) oncologic and functional evaluation following IRE as a primary treatment in 229 patients (International Society of Urologic Pathologists [ISUP] grade 1–4).^196^ The long-term follow-up confirmed earlier findings that IRE provides acceptable local and distant oncological control with lower loss of continence and potency than radical treatments.

Radiotherapy is a well-established therapy for PCa; however, one in five patients recur with significant disease, forming a difficult-to-treat patient sub-population. Recently, IRE has been evaluated in patients with recurrent PCa, specifically following prostatectomy and radiotherapy.^197–199^ Mid-term oncological and safety results demonstrate that IRE can be delivered safely to ISUP 1–5 recurrent patients, with similar in-field oncologic responses to *in situ* treatment.^197^

Dong *et al*. were the first to demonstrate the feasibility of tumor ablation using H-FIRE in humans.^115^ They treated 40 PCa patients using a 5 μs pulse width without ECG synchronization and with moderately lower muscle relaxants than conventional treatments. No muscle contractions or abnormalities were observed during H-FIRE delivery, with all patients able to move ~10 hours after treatment. Lesions were clearly visible on MRI at 4 weeks post-treatment. At a median follow-up of 6 months, no major complications were experienced, with sexual function and urinary continence preserved in all patients. A recent multi-center nonrandomized prospective clinical study treated 109 patients with low (n = 27) and intermediate (n = 82) risk PCa using an unspecified H-FIRE waveform.^116^ One hundred patients underwent a 6-month biopsy, with clinically significant prostate cancer in the treatment zone and out of the treatment zones for 1 and 5 patients, respectively. Urinary continence was maintained in 99.1% of patients, and emergent sexual dysfunction was experienced in 9% of patients.

## Pancreas (PC)

Pancreatic cancer is currently the 3^rd^ deadliest malignancy and possesses an insidious prognosis due to its surreptitious progression, with over 80% of patients unfortunately presenting stage III locally advanced pancreatic cancer (LAPC) or metastatic disease at diagnosis. Poor outcomes for LAPC are attributed to diffuse cancer infiltration, the sclerotic and immunosuppressive tumor microenvironment, and significant involvement of sensitive structures. This precludes surgical resection in > 80% of patients. The intervention of unresectable PC consists of chemoradiation, which has not meaningfully increased survival, with a median overall survival of 9.3–11.8 months after diagnosis.^200,201^ IRE provides perhaps one of the largest benefits to patients with LAPC, and numerous clinical evaluations are published yearly, demonstrating its safety and efficacy. Further, multiple studies have evaluated IRE to treat margins after pancreatectomy in borderline resectable pancreatic cancers (BRPCs), termed margin accentuation (MA), when negative margins are not expected.

Martin *et al*. and Narayanan *et al*. published the first clinical series on the treatment of PC using IRE.^156,202^ Martin *et al*. treated 27 patients with IRE either *in situ* (n = 19) or for MA following surgical resection (n = 8). They achieved 100% ablation of the primary tumor evaluated at the 90-day follow-up. Nine patients experienced 18 complications, with most being potentially associated with the open surgery approach and 4 being possible devicerelated complications. In parallel, Narayanan *et al*. treated 11 patients with LAPC and 3 with metastatic disease using a percutaneous approach. Ten of the 11 LAPC patients were still alive at 14 months post-treatment, but the 3 metastatic patients did not benefit from IRE with a median overall survival of 4 months. Contrast-enhanced CT immediately and 24 hours after treatment showed that vascular patency was preserved in all patients. Martin *et al*. subsequently treated 200 Stage III LAPC patients treated with either *in situ* (n = 150) or for MA following surgical resection (n = 50).^203^ All patients had initially undergone induction chemotherapy, and 52% were additionally given chemoradiation therapy for a median of 6 months before IRE. At a median follow-up of 29 months, 58 patients developed recurrences (6 local recurrences) with a median progression-free survival of 12.4 months. MA had a higher median overall survival than IRE alone (28.3 *vs*. 23.2 months). Twenty patients (40%) experienced 49 complications in the MA group, and 54 patients (36%) experienced 100 complications in the *in situ* group, with the most common complications being gastrointestinal complaints. Ten severe complications were experienced after treatment. The same group published their results on another prospective multi-institutional assessment with 152 additional patients treated.^67^ *In situ* IRE was successfully delivered to all patients with tumors ranging from 1 to 5.4 cm in diameter with a median follow-up of 19 months. There were 9 local recurrences and 27 distant recurrences, resulting in a median progression-free survival of 22.8 months and a median overall survival of 30.7 months. Nineteen patients experienced severe adverse events, with the most common complications being gastrointestinal or hepatic related. In both studies, the liver was the most common site of distant recurrence.

Many clinical studies have evaluated IRE following inductive chemotherapy. A randomized trial demonstrated the additive effect of IRE with or without chemotherapy.^204^ Specifically, combinatorial treatment patients had higher OS (20.3 *vs*. 16.2 months). Similarly, the PANFIRE-2 trial found IRE following induction chemotherapy provided a benefit to OS (17 *vs*. 12.4 months).^140^ A recent prospective randomized clinical trial compared the safety and efficacy of IRE (n = 34) to MRI-guided stereotactic ablative body radiotherapy (SABR, n = 34) following induction FOLFIRINOX.^205^ There were no differences in OS (12.5 *vs*. 16.1 months), PFS (9.5 *vs*. 8.5 months), or number of complications. Distant tumor-free survival was higher following IRE (13.2 *vs*. 8.5 months), but this could be due to a higher percentage of patients receiving adjuvant therapy following IRE. He *et al*. analyzed the SEER and SYUCC databases to compare the efficacy and long-term safety of IRE (n = 206) following induction chemotherapy against chemotherapy alone (n = 3444)^206^ and found that IRE following induction chemotherapy had a higher OS (18 *vs*. 8 months) and PFS (7.7 *vs*. 4.1 months). Recently, Suraju *et al*. compared resection (n = 40), MA (n = 13), *in situ* IRE (n = 14), and unresected (n = 35) in BRPC and LPAC patients who received neoadjuvant chemotherapy.^236^ Despite having a higher number of patients with LAPC in the MA group, they experienced a non-significantly higher OS and PFS compared to resectable patients; the median OS from diagnoses were 30 months for MA, 28 months for *in situ* IRE, 27 months for resection, and 14 months for the unresected group. Neoadjuvant chemoradiation, IRE, and resection were independently associated with decreased risk of mortality, and IRE with an open approach had fewer severe complications than pancreatectomy.

## Liver

Liver cancer is the fifth most fatal malignancy globally, with hepatocellular carcinoma (HCC) comprising over 80% of primary liver tumors.^207^ Additionally, the liver is a frequent site of metastasis, especially from colorectal cancer; at least 25% of colorectal cancer patients develop liver metastases (CRLM), accounting for a substantial proportion of secondary liver tumors.^208^ Standard treatment approaches for HCC and CRLM, including chemoradiation and surgical resection, are often limited, and up to 80% of patients are deemed ineligible for resection due to tumor burden, anatomical location, or proximity to critical structures.Following hepatectomy, critical structures like the single remaining portal vein, central bile duct, and one or two major hepatic veins limit further resection, as removal or damage to these could compromise liver function. If further resection of these structures is not feasible, then focal ablation offers an effective treatment, but thermal ablation strategies are limited due to the associated “heat sink” effects and potential damage to critical structures.

Thus, IRE has been an increasingly effective method for treating tumors near these structures.^155,209^ Ma *et al*. demonstrated that percutaneous IRE is a safe and effective treatment for HCC abutting the diaphragm.^210^ They successfully ablated 36/39 tumors with no major complications and achieved a median 20.4 months to local tumor progression. The COLDFIRE-I ablate and resect clinical trial demonstrated the feasibility and safety of IRE to treat CRLM in 10 patients.^211^ The subsequent COLDFIRE-II trial further demonstrated the efficacy and safety of IRE in 51 patients with a total of 76 CRLMs.^237^ The 1-year local-progression-free (LPF) rate was 68%, and following repeated procedures in 8 patients, local control was achieved in 37/50 (74%) patients. The median overall survival from treatment was 32 months. Fruhling *et al*. further demonstrate that IRE was a safe ablation modality in 149 patients with HCC (n = 53) and CRLM (n = 71) when other treatment options are unsuitable.^212^ At 12 months, they achieved local ablation success of 40.3% in HCC patients and 25.4% in CRLM patients. This translated to a median OS of 35 months and 27 months for HCC and CRLM patients, respectively. Three patients experienced severe complications, with one death due to thromboembolism. In a subsequent analysis of the patient population, they found that smaller decreases in resistance and larger tumor sizes were associated with earlier recurrence in CRLM but not HCC patients.^213^

In an evaluation of IRE as a salvage treatment, Hitpass *et al*. demonstrated that IRE is a safe option when resection and thermal ablation are unsuitable.^84^ All tumors were located adjacent to the sole remaining intrahepatic blood vessels and bile ducts, but IRE was successfully delivered with a 5 mm margin in 31/32 lesions across 23 patients, with one incomplete ablation. The local progression-free rate was 64% and 57.4%, and the intrahepatic progression-free rate was 36.4% and 19.5% at 12 and 36 months, respectively. Altogether, five patients were tumor-free at the last follow-up. No vessel injury or thrombosis was observed, and only minor complications occurred, including moderate segmental cholestasis, which spontaneously resolved. Recently, Narayanan *et al*. confirmed that IRE is a safe and viable option for the treatment of unresectable CLRMs close to the portal and hepatic veins, inferior vena cava, bile duct, and gallbladder.^214^ They achieved a median OS of 40.4 months with only minor complications. In a recent randomized non-inferiority clinical trial, Zhang *et al*. compared IRE (n = 78) to radiofrequency ablation (RFA) (n = 78) for the treatment of malignant liver tumors.^215^ They demonstrated that IRE was not inferior to RFA, with comparable tumor ablation rates (94.9% *vs*. 96%), similar complication rates, and similar 6-mo recurrence rates (13.3% *vs*. 19.7%) between IRE and RFA. In a direct comparison of IRE to RFA and MWA in a propensity score-matched population of early HCC, Wada *et al*. found 2-year local tumor progressions of 0%, 45%, and 25% for IRE, RFA, and MWA, respectively.^216^

A majority of HCC develops in patients with underlying pathologies, and the possibility of damaging diseased hepatic parenchyma (e.g. Child-Pugh B/C) has the associated risk of severe liver failure and mortality.^217^ Bhutiani *et al*. compared the tolerability and efficacy of IRE and microwave ablation for treating HCC patients with moderate Child-Pugh B liver dysfunction.^218^ They found that both modalities had comparable success rates, but IRE was better tolerated with a significantly lower length of stay and 90-day readmission rate.

## Kidney

Small renal cell carcinoma (RCC) has traditionally been treated with surgical resection, with radical nephrectomy being the most common treatment. IRE has yet to be fully established for the treatment of renal tumors, but it may be considered when surgical resection or thermal ablation is not an option. Thomson *et al*. treated 7 patients with RCC using IRE.^95^ Transient hematuria was observed in two patients with treatments near the center of the kidney, which resolved in under 24 hours. Followup CT at 3 months confirmed successful ablations in 71.4% (5/7) of patients, with the other 2 receiving a second IRE procedure. The first large cohort of patients with renal tumors treated with IRE was reported by Trimmer *et al*., in which 20 patients with T1a renal carcinoma (n = 13), indeterminate masses (n = 5), or benign masses (n = 2) underwent CT-guided IRE.^86^ All ablations were initially technically successful, as verified with ce-CT, but two patients required salvage therapy at 2 weeks due to incomplete ablation. All 15 patients imaged at 6 months had no evidence of recurrence, and only one patient was observed to experience recurrence at 1 year after IRE.

Despite initial data supporting the feasibility and safety of IRE, a few clinical studies have found suboptimal short- and mid-term disease control. Canvasser *et al*. found that the initial treatment was successful in 93% (39/42) of tumors, but the 2-year local-recurrence-free rate was 83%^89^, which is unfavorably compared to contemporary local-recurrence free rates of >97% for partial nephrectomy of tumors < 3.0 cm. Further, the first prospective Phase II clinical trial (IRENE) found “complexities in the overall procedure”.^92^ All tumors were resected after treatment to assess the lesion. Four patients had no residual tumor, while 3 had microscopic residual tumor due to incomplete ablation. Dai *et al*. found similar results in a retrospective study of 47 patients with 48 tumors, with 45.8% (22/44) being biopsy-proven RCC.^219^ At a median follow-up of 50.^4^ months, their 5-year local recurrence-free rate was 81.4% in biopsy-confirmed RCC patients and 81.0% in all patients.

None of the studies observed major complications, supporting the safe initial use of IRE for RCC. While the safety profile after IRE is compelling, if it is concluded that IRE does not present a significant advantage over conventional therapies, patient selection for IRE could include those with central renal tumors near blood vessels and collecting systems in which the nonthermal mode of ablation can be exploited. Min Wah *et al*. evaluated the safety and efficacy of CT-guided IRE in 26 patients with 30 biopsy-proven RCCs near vital structures of the kidney.^96^ Nearby structures included the colon (n = 11), ureter (n = 11), and renovascular pedicles (n = 7). They specified that the initial technical success of 73.3% was due to an early operator’s learning curve, and 7/8 of the residual tumors were treated with CA to achieve a technical success rate of 97%. They state that one patient was not retreated due to an unexpected stroke at 4 months post-IRE. The 2- and 3-year recurrence-free survival was 91% for both time points. Six patients experienced minor complications, and 1 patient experienced a major complication (Clavien-Dindo III), as the patient developed post-proximal ureteral stricture that required long-term retrograde ureteric stenting.

## Lung

Lung cancer is the deadliest and most prevalent cancer globally, with few curative treatment options. Central tumors near the central bronchial structures and large blood vessels are especially challenging to treat with surgical resection and thermal ablation modalities. IRE can potentially spare critical structures, but current oncological outcomes are lacking.

Thomson *et al*. treated 1 patient with 1 non-small-cell carcinoma and 3 patients with 5 colorectal lung metastases.^95^ None of these patients treated with IRE had a satisfactory tumor response, and they all presented with progressive disease when assessed by the 3-mo time point. A biopsy from one of the patients showed coagulative necrosis in a portion of the tumor with viable cancerous tissue at the margin of the treated lesion. All four patients experienced transient ventricular arrhythmia, one patient presented transient supraventricular tachycardia, and one patient required cardioversion as a response to atrial fibrillation. Pneumothorax was observed in two out of the four patients which resolved spontaneously. Usman *et al*. reported on the use of IRE to treat two patients with lung neoplasms that had been previously deemed unresectable.^98^ One of the patients presented with an increase of the right suprahilar mass with ce-CT, suggesting tumor growth reported 2 months after the procedure. Moderate parenchymal hemorrhage was observed during the procedure, and at the 9-month follow-up, it was suggested that the tumor had invaded the trachea. The cancer continued to progress, and the patient succumbed to the disease within a year post-IRE. The other patient was reported to still be alive 2.5 years after the procedure, with no major complications described. The authors explain that challenges still remain with using IRE to treat lung tumor masses due to the heterogeneity, geometry, and low density of lung tissue. It is clear that further research is needed to optimize IRE treatment of lung cancer through collaboration between engineers and clinicians. It can be argued that these studies were limited because the probes themselves were not designed for lung treatments, and thus, surgical probes need to be tailored for this particular application.

Kodama *et al*. determined that electroporation applied through an endobronchial catheter is a feasible technique for the treatment of parabronchial tumors in a pig lung tumor model.^220^ The ablations measured on gross pathology were significantly smaller than the treatment-related changes measured on CT, contrasting observations in other organ systems. Using FEM, they predicted EFs sufficient to induce irreversible electroporation (500– 2000 V/cm) within a 1 cm circumference around the probe, which was reflected by extensive ablations seen in gross histology. However, large blood vessels and airways significantly affected the EF distribution, reducing the local EF in portions of the tumor below the lethal EFT. Lastly, they found that electroporation does not affect the patency of the treated bronchi.

## Cardiac

Catheter-based PFA is emerging as a promising alternative to thermal techniques (RFA & CA) in treating cardiac arrhythmias due to the better safety profile and similar efficacy.^221,222^ The rapid success of PFA in the clinic has led many research groups and companies to develop their own probes and electroporation systems (Figure 8), often keeping technical details and treatment parameters secret. Direct electric currents were first used to treat cardiac arrhythmias in the 1980s; however, the continuous application of the EF caused electrical arcing, barotrauma, and proarrhythmic effects. Lavee *et al*. were the first to utilize IRE for atrial ablation in 5 pigs, which mitigated the previous complications experienced with direct current applications^124^ and achieved sharp transmural with no evidence of thermal damage. Subsequently, preclinical and clinical studies have demonstrated that PFA selectively ablates cardiac tissue while minimally affecting peri-atrial tissue, such as the esophagus and phrenic nerve^223^, and lowers the risk of pulmonary vein stenosis compared to thermal ablation. Recently, the results from multiple large clinical trials have been released.

The first and most studied PFA catheter is the multi-electrode pentaspline catheter.^122^ The Impulse, PEFCAT, PEFCAT2, and PersAFONE trials demonstrated the initial feasibility and safety of this catheter for treating paroxysmal and persistent AF in relatively small cohorts.^224^ Recently, the MANIFEST-PF^117^ and MANIFEST-17k^225^ clinical trials provide compelling safety and efficacy results in larger patient cohorts and across more centers. The MANIFEST-PF trial included 24 centers and 1,758 patients to determine the acute effectiveness and safety of PFA and found that PFA achieved complete acute pulmonary vein isolation in 99.9% of patients on immediate electroanatomical mapping. The 1-year recurrence rates were 31% for the total cohort, 27% for paroxysmal AF, and 42% for persistent AF. The MANIFEST-17k trial evaluated the safety of PFA across at 106 centers across 20 countries in 17,642 patients with paroxysmal (57.8%) and persistent (35.2%) AF. At a median of 15 months follow-up, no esophageal damage, pulmonary vein stenosis, or persistent phrenic nerve palsy were reported. Major complications were reported in 0.98% of patients, with the most common being pericardial tamponade (0.36%), vascular events (0.30%), stroke (0.12%), hemolysis-related acute renal failure (0.03%), and death (0.03%). Two of the deaths (0.01%) were procedure-related from irreversible neurological damage; post-procedural brain MRI was performed in 96 asymptomatic patients to determine the rate of silent cerebral lesions (SCLs), of which 9.4% of patients showed abnormalities. Further, the recent ADVENT trial demonstrated the non-inferiority of PFA using the pentaspline catheter in a randomized, single-blind prospective comparison to conventional thermal ablation (RFA or CA) in 707 paroxysmal AF patients^221,222^ evaluating the safety and 1-year recurrence rates of pulsed-field ablation against thermal ablation (RFA or CA). Urbanek *et al*. found similar results in 400 patients and achieved similar 1-year success rates between CA and pentaspline PFA in both paroxysmal AF (83.1% CA *vs*. 80.3% PFA) and persistent AF (71% CA *vs*. 66.8% PFA).^126^

The PULSED AF pivotal trial evaluated the circular-lasso-type 9-electrode catheter in 150 paroxysmal and 150 symptomatic persistent AF patients.^125^ They achieved 100% acute pulmonary vein isolation rates for both groups, but at the 90- day follow-up, the recurrence rate was already 30.5% and 37.7% for the paroxysmal and persistent AF groups, respectively. The 1-year recurrence rates did not increase much from the 90-day rates, with 33.8% for the paroxysmal AF and 44.9% for the persistent AF patients. Two severe adverse effects occurred due to treatment (0.7%): one cerebrovascular accident occurred the same day as treatment and one pericardial effusion that required draining.

The SPHERE PER-AF trial is a randomized, 2-arm prospective study evaluating a large-tip catheter dual PFA and RFA ablation system against a control RFA system.^226^ They found that PFA had significantly lower energy application times, transpired ablation times, and skin-to-skin procedural times. At a 1-year follow-up, 73.8% and 65.8% of patients were arrhythmia-free for the large-tip catheter and control system, respectively, with no major complications observed in either group.

The insPIRE and admIRE trials investigated the safety and efficacy of using a variable-loop circular catheter (VLCC).^227,228^ The inspIRE trial investigated the safety and efficacy of the VLCC in 226 patients with paroxysmal AF. The 12-month freedom from symptomatic arrhythmia was 79%. Pre- and post-treatment MRI imaging detected SCLs in 4 of the first 6 patients. After adjusting treatment to include a 10-second pause between PFA applications and strictly adhering to the anticoagulation regimen, SCLs were found in 4 of the remaining 33 patients. All the SCLs were asymptomatic and resolved spontaneously. The VLCC can be used for guidance, stimulation/recording of cardiac signals, and applying PFA, so the admIRE trial investigated the use of the VLCC for real-time non-fluoroscopic procedural guidance and lesion indexing in 277 patients with paroxysmal AF. They achieved 97.5% success on first-pass per vein isolation, with 100% of veins ultimately isolated. At 12 months, they found similar efficacy to patients treated without fluoroscopy (75% *vs*. 72.7%), demonstrating that treatments can be delivered without fluoroscopy, which can potentially speed up procedures, minimizing procedure-related complications and exposure to X-rays.

Collectively, these results indicate that H-FIRE is a safe and effective method for pulmonary isolation, but high acute pulmonary isolation rates have not necessarily translated to long-term freedom from disease. Nevertheless, PFA has similar, if not slightly better, efficacy than thermal ablation, but currently, methods are still needed to generate deeper and wider transmural lesions to prevent recurrence.

Multiple preclinical and early clinical evaluations have also demonstrated the feasibility of PFA for the treatment of ventricular arrhythmias (VAs).^229–231^ VAs pose a unique challenge due to the thickness of the tissue and frequent scar tissue, making it challenging to develop deep lesions. PFA is indicated to better penetrate through scar tissue^231–233^, allowing for treatment of tissue that other focal ablation therapies cannot reach and for redo ablations. Peich *et al*. evaluated focal PFA in 21 patients with ventricular premature complexes and 23 partients with scar-related ventricular tachycardia.^234^ Using the highest energy setting (25A), they achieve 81% and 52% success for the premature complex and tachycardia patients, respectively, at a mean follow-up of 116 days.

## Concluding remarks

It has almost been 300 years since the earliest recording of electrically mediated tissue damage by Jean-Antoine Nollet in 1754. He observed the formation of red spots, presumably due to IRE, following the application of high voltages to human and animal skin. Only 20 years ago was IRE again described as a viable option for controlled tissue destruction. In such a short period, it has significantly impacted the treatment of soft tumors and cardiac tissue. However, there are still multiple areas of improvement:

(1) Factors influencing electroporation at the cellular and tissue level are still not fully understood, and there is still a large gap in knowledge on the precise mechanisms of cell death following different PFA procedures. PFA is unique compared to every other focal therapy, and understanding genetic and proteomic changes following treatment is paramount for developing synergistic therapies.
(2) Accordingly, the dynamics of tumor microenvironmental changes following PFA have only recently started being investigated.
(3) Electroporation-dependent tissue properties for many tissues and tumors are not available, and there are currently no guidelines on appropriate methods for gathering and validating data. This limits confidence in computational models for predicting ablation outcomes before treatment.
(4) Inserting and maintaining multiple probes is the most technically challenging and time-consuming aspect of IRE treatments. Improved methods for delivering PEFs will presumably help increase the adoption of PFA and decrease operating room times.
(5) While ablations can be measured soon after treatment, there are no clinically ready methods for real-time ablation progression or temperature monitoring. The lack of real-time feedback can lead to unnecessary thermal damage and avoidable complications.
(6) Due to the multifaceted nature of PFA, optimized waveforms for oncology and cardiology have yet to be developed.

Therefore, it is important for industry, clinicians, and researchers to work together to allow for independent analysis and validation of data. If clinicians are aware of the capabilities and limitations of PFA procedures, tissues that were once considered untreatable and unresectable may now find a legitimate contender with IRE.
